# The changing epidemiology of paediatric childhood asthma and allergy in different regions of the world

**DOI:** 10.3389/falgy.2025.1584928

**Published:** 2025-04-30

**Authors:** D. J. Adamko, Kyla J. Hildebrand

**Affiliations:** ^1^ Division of Respiratory Medicine, Department of Pediatrics, University of Saskatchewan, Saskatchewan, SK, Canada; ^2^ Division of Immunology, Department of Pediatrics, University of British Columbia, Vancouver, BC, Canada

**Keywords:** asthma, allergy, epidemiology, inborn error immunity, primary atopic disorders

## Abstract

Allergic disorders encompass a variety of conditions including asthma, atopic dermatitis, food allergy, allergic rhinitis, and eosinophilic esophagitis. These atopic disorders are connected via an abnormal host immune response to the environment. A series of longitudinal cross-sectional studies conducted over the past 3 decades have reported on the epidemiological trends that contribute towards the development of pediatric asthma and allergic disease. Infant birth cohort studies assessing the microbiome have offered clues as to the underlying biological mechanisms and basis for allergic disease. Why this abnormal immune response is occurring is the basis of decades of research and the reasons for this chapter. Our understanding of the biology of the immune system has increased exponentially with the advances in genomic testing, providing further opportunity for targeted treatments and more importantly, primary prevention of atopic disease.

## Introduction

Asthma and allergic diseases are the most common chronic pediatric conditions and are leading healthcare costs. In 2013, direct costs of pediatric asthma were US$5.92 billion, with the average annual costs per child ranging from US$3,076 to US$13,612 (1). Allergic disorders encompass a variety of conditions including asthma, atopic dermatitis, food allergy, allergic rhinitis, and eosinophilic esophagitis. Allergic disease develops in a time-based sequence, often referred to as the ‘atopic march’: beginning with atopic dermatitis and food allergy in infancy, to the development of asthma and allergic rhinitis in later childhood or beyond (2, 3). Severe forms of atopic diseases are a manifestation of immune dysregulation and reflect a new category of inborn errors of immunity collectively defined as primary atopic disorders (4). These allergic conditions are connected via an abnormal host immune response to the environment. For reasons that are currently unknown, exposure to common environmental factors like animal dander, certain foods, inhaled molds or pollens are not tolerated but instead generate a T Helper type 2 (T_H_2)-like immune response driven by proinflammatory cytokines such as interleukin (IL)-4, IL-5 and IL-13 which activate effectors cells such as mast cells and eosinophils, thus driving inflammation. Classically, the diagnosis of allergic disease is established clinically based on the presence of effector cells like eosinophils or immunoglobulin E (IgE)-mediated activation of mast cells and/or basophils. These immune cells promote an allergic response leading to symptoms of asthma such as paroxysmal cough, dyspnea, and wheezing; for allergic rhinoconjunctivitis symptoms of rhinorrhea, angioedema and conjunctivitis, and urticaria, flushing and possibly hypotension in the setting of anaphylaxis (5).

## Understanding the biology driving inflammation is a key goal for research and prevention

Environmental exposures vary in an urban vs. rural setting, which seems to factor into the development of allergic disease. The term ‘hygiene hypothesis’ was coined in 1989 by Strachnan reflecting an observational theory that children with fewer viral infections in early life were more likely to develop allergic rhinitis (6). Stiemsma et al. summarized the evolution of the immune dysregulation associated with the hygiene hypothesis including the early report of low incidence of rheumatoid arthritis in Western Nigeria where malaria exposure is frequent (7) and how experimental theories have expanded the hygiene hypothesis to also include commensal and symbiotic intestinal microbes (8). One of the earliest studies assessing the role of farm exposures was described by Martinez and Holt by comparing two cross-sectional studies in Europe and concluded that children who lived on farms are exposed to a more diverse range of microbial agents and develop less allergic disease as demonstrated by reduced prevalence of asthma and atopy (9). Stein et al. demonstrated that even among genetically similar populations such as the Amish and Hutterite communities in the United States, environmental exposures as it relates to differing farming practices elicits varying innate immune responses. Amish farmers use a traditional practice with exposure to environments rich in microbes, whereas Hutterites apply industrialised technology. None of the Amish children studied had asthma in contrast to 20% of Hutterite children (10). However, the impact of rural exposures on asthma is complex and in some cases under-diagnosis of asthma may be occurring in these rural/remote communities (11). New research assessing the modality or route of exposure (inhaled, ingested) and effect on the varying tissue microbiomes (respiratory, intestinal, cutaneous) are emerging as a key question in our understanding of causal mechanisms (12). Early life exposures to inflammation generate innate immune responses that vary within and across populations (13). The discovery of T regulatory cells and innate lymphoid cells add to our understanding of the complexities of the immune system in atopic disease development beyond Th1 vs. Th2 imbalances (14). The increased prevalence of allergic disease has been attributed in part to epigenetic mechanisms such as DNA methylation, whereby environmental effects create biochemical changes of transcriptionally relevant genetic information without altering the nucleotide sequence of the genome (15). Evolutionary life history theory considers the relative cost and effectiveness of different immune responses: innate immune responses are lower cost up front however are imperative for novel infections; acquired immune responses are most effective against secondary infections due to antigen-specific antibodies however require nutritional abundance (16). Socioeconomic factors, including access to healthcare, complicate the relationship of location and allergy.

Nutrition also plays a role in homeostasis between the balance of Th_1_ and Th_2_ immune responses. Micronutrient deficiencies may contribute to atopic diseases: iron, zinc, vitamins A and D deficiencies are drivers of atopic diseases. Children with allergic diseases in the US and China are up to 8 times more likely to be anemic compared to children without allergic disease. Dietary restrictions due to food allergy can also lead to micronutrient deficiencies (17). Gut microbiome studies of semi-nomadic gatherer/hunter people of the Yanomami has been found to be distinctly more diverse when compared to westernized counterparts. Indigenous diets contain high-fiber plant products rich in inulin, which has been shown to stabilize gut homeostasis via short chain fatty acids that inhibit pro-inflammatory cytokines when absorbed by gut epithelial cells (18).

## Epidemiological trends of asthma and atopic disease among children and youth

Asthma is one of the most common chronic respiratory conditions of childhood worldwide, but regional differences exist. Several large population-based and cohort studies have shaped our understanding of asthma and allergic disease worldwide, providing early clues into the factors impacting the epidemiology of allergic disease and asthma worldwide including climate change, pollution, nutrition, birthing patterns, use of antibiotics early in life, and environmental exposures including viral infections.

In North America, data from the Center for Disease Control in the United States in 2019–2021 reported the prevalence of asthma among children less than 18 years was 6.5%. Prevalence was higher among youth 15–17 years (9.5%), in Black/African American children (11.6%), in Puerto Rican children (17%), and in those with the lowest household incomes (10.2%). Data from the National Institute of Health reported the prevalence of childhood asthma was 8.4% in 2017 declining to 5.8% in 2020, however the authors caution that a newer data collection strategy, coupled with the COVID19 pandemic reducing the number of viral respiratory tract infections, may be contributing factors to the declining prevalence (19).

The International Study of Asthma and Allergies in Childhood (ISAAC) is the largest study examining the prevalence and severity of asthma symptoms, allergic rhinitis and eczema among children in different parts of the world. The ISAAC studies advanced our understanding of the epidemiology of pediatric asthma, and led to the exploration of environmental factors that might contribute to the asthma and the atopic march at a population level. This cross-sectional study consisted of three phases. ISAAC Phase I (1994–1995) involved the assessment of prevalence and severity of asthma and allergic disease and included 700,000 school-age children from 56 countries using simple core questionnaires that were easy to implement regardless of health care resources. Phase II investigated possible etiological factors as suggested by Phase I; and Phase III (2011–2003) was a repetition of Phase I to assess trends in prevalence over time. There were two age groups of children studied: 6–7- and 13–14-year-olds. The Phase I study found significant differences in the prevalence of asthma between countries: In the 13–14-year-old age group, the prevalence of wheeze in the preceding 12 months ranged from 2.1 in Indonesia to 31.2% in the United Kingdom (UK) and 4.1 in Indonesia to 32.1% in Costa Rica in the younger age group. Prevalence was higher in countries that were English speaking or Latin America. This study had three countries with 14–15 study sites each, however the prevalence of asthma varied from low in India, moderate in Italy and high in the UK, suggesting discrepancies were most consistent between countries compared to within countries (20).

ISAAC also reported prevalence results for allergic rhinitis and atopic dermatitis. Allergic rhinitis symptom prevalence was reported between 0.8% and 14.9% in the 6–7-year-olds and from 1.4% to 39.7% in the 13–14-year-olds. This variation in reporting of allergic rhinoconjunctivitis symptoms highlighted differences in how allergic disease symptoms are labelled and reported at a population level (21). For atopic eczema, the ISAAC study reported that the highest prevalence rates were seen in countries where asthma rates were not similarly elevated including Scandinavia and African countries (22). The discordance in prevalence for atopic diseases within a country reflects a need for further research as to why some countries seemingly follow an ‘atopic march’ and others may not.

ISAAC Phase III study included approximately 1,200,000 children from 233 centers in 98 countries. Results found that the global prevalence of current asthma, rhinoconjunctivitis and eczema in the 13–14-year age group was 14.1%, 14.6% and 7.3%, respectively. Consistent with results from the Phase I study, The Phase III study demonstrated a wide variability in the prevalence and severity of asthma, rhinoconjunctivitis and eczema depending on the regions. Additionally, the Phase III study further established that the prevalence of allergic disease is high in non-affluent centres with low socioeconomic conditions. This suggests local environment characteristics play a key role in determining the differences in prevalence between one place and another (23).

To improve our understanding of asthma symptoms in younger children, the International Study of Wheezing Infants (EISL) was performed as a multicenter, cross-sectional study assessing the prevalence of recurrent wheezing and related risk factors in infants during the first year of life. This study revised the ISAAC questionnaire and validated it for infants less than 12 months of age and administered the survey between 12 and 15 m of age. In 2005, 30,093 children participated from 17 centers [25,030 in Latin American (LA) countries in 12 sites; and 5063 from 5 European (EU) sites]. Of the results reported, symptoms of a viral infection in the first 3 months of life were the most consistent factor associated with a shorter time to first episode of wheeze (24).

More recently the Global Asthma Network (GAN) Phase 1 study published results from a cross-sectional study striving to report on global surveillance of asthma prevalence, severity, management and risk factors. This was the first study since ISAAC that aimed to assess global asthma prevalence over a 27-year period among school age children. Results include a decrease in prevalence for severe asthma symptoms in adolescents however there was an overall increased report of ever having asthma symptoms and night cough in both adolescents and school age children. Key findings of the GAN study indicates that the overall prevalence of asthma remains stable, however 1 in 20 children worldwide has severe asthma (25).

Lastly, the Environmental Influences on Child Health Outcomes (ECHO) study assessed 5,809 children from 10 of the 12 cohorts from the Children’s Respiratory and Environmental Workgroup (CREW) birth cohort consortium. This study assigned census-derived tract-level data for socioeconomic, demographic, and housing variables. Study findings included Black and Hispanic children had higher rates of asthma and asthma morbidity compared to White children; Black and Hispanic children in this study resided in communities with economic deprivation at a disproportionate rate compared to White children. Applying census data to the CREW cohorts, the authors reported environmental characteristics at birth relating to population density and poverty were associated with an elevated risk of asthma incidence. Black and Hispanic children were at higher risk than White children for developing asthma regardless of neighbourhood, further linking race, ethnicity and neighbourhood factors with onset of asthma (26).

## The role of viruses in the development of asthma

Viruses are important factors in the environment which the host must respond to. This response can be important in the development of asthma and the diagnosis of asthma (27). Numerous studies have shown that infants (<2-year-olds) with a more severe response [i.e., hospitalized with Respiratory Syncytial Virus (RSV) bronchiolitis] are at increased risk of developing later asthma and in some cases even allergic disease (28–32). In addition, there are data that other viruses which cause hospitalization at this young age (i.e., human metapneumovirus) can also increase the risk of later asthma and allergic disease (33). It is also well described that common cold viruses like Rhinovirus are a key trigger for an asthma exacerbation in all ages. People that present to urgent care with Rhinovirus are much more likely to have a diagnosis of asthma (34). The host response to these common viruses is critical to our understanding of allergic disease and asthma (35). For example, there is excellent data *in vivo* and *in vitro* that typically allergic effector cells like eosinophils can be triggered by viral infection to release mediators and induce airway hyperresponsiveness (36, 37). RSV has unique characteristics that could attract allergic effector cells to the airway (38). Overall, the reason why people with allergic inflammation respond so poorly to common colds is an important factor in our understanding of allergic airway disorders.

## The role of the microbiome: expanding understanding of genes and environment on development of allergic disease

The Canadian Healthy Infant Longitudinal Development (CHILD) Study is a longitudinal birth cohort study that aims to advance knowledge about the genetic and environmental determinants of atopic diseases including asthma, allergy, allergic rhinitis and eczema. This study recruited 3,500 pregnant women who gave birth between 2009 and 2012 from 4 provinces and followed the infants prospectively. One of the first published reports from this cohort study reported on the gut microbiome of 24 healthy infants and correlated with mode of delivery and feeding status (breastfed or formula fed). Infants born by elective caesarean section had low bacterial diversity and richness. This study advanced understanding of infant gut microbiota and offered new evidence correlating to mode of delivery (39). Another key finding from the CHILD study is the impact of the timing of systemic antibiotics administered within the first year of life are associated with risk of atopic dermatitis [adjusted odds ratio (aOR) = 1.81; 95% CI: 1.28–2.57; *P* < 0.001]. Additionally, antibiotics in the first year of life were linked to infant gut microbiome disruption and elevated atopic dermatitis risk. This study also identified key gut microbiome components that could potentially be used to predict and possibly prevent the onset of atopic dermatitis (40). Antibiotic stewardship programs have successfully reduced the use of antibiotic prescriptions in infants and children: a systematic review of 113 studies assessing antimicrobial stewardship programs in children 0–18 years, 79.6% of the studies showed a significant reduction in inappropriate antibiotic prescriptions (41). A Canadian population level study coupled with a prospective cohort analysis found that declining rates of asthma diagnosis at age 1–4 years was associated with decreased antibiotic use in infancy (age < 1 year). Asthma incidence increased by 24% with each 10% increase in antibiotic prescribing (adjusted incidence rate ratio 1·24 [95% CI: 1·20–1·28]; *p* < 0.0001) (42). Birth cohorts from around the world continue to collect key information and data, providing clues on mechanisms of disease for the development of asthma and atopic disease, and offer strategies towards primary prevention at an individual and population level.

## Severe atopy: the tip of the iceberg for uncovering inborn errors of immunity (IEI)

While the environment is important, family history and a genetic basis for disease is well-described. Inborn errors of immunity are a group of disorders caused by damaging germline variants in single genes. The International Union of Immunological Societies (IUIS) has curated and maintained an updated list of inborn errors of immunity since the 1970s. In the United States the incidence of IEIs is 6 per 10,000 individuals (43). Although once considered a rare group of diseases, more recently our understanding has shifted to recognize that collectively these disorders are more common and important for allergic diseases. In the 2024 summary, the IUIS listed 555 IEI’s and 17 phenocopies due to genetic variants in 504 different genes (44). Severe atopy including atopic dermatitis, allergic asthma and food allergy are a well described presentation for a group of IEIs categorized as primary atopic disorders (PADs), a term first coined in 2018 to delineate this distinct group. PADs are a group of heritable monogenic allergic disorders, often characterized by severe, early-onset, and co-existent allergic conditions such as atopic dermatitis, food allergy and allergic asthma (45). One of the challenges of PADs is that environmental and host factors can contribute to the heterogeneity of the clinical presentation and severity of disease, requiring clinicians to have a high index of suspicion for IEI, particularly in the absence of infections (46). Our understanding of the biology of these conditions has laid the groundwork for therapies for some of the most common allergic disorders affecting large portions of the population.

## Conclusion

Allergic disorders affect a significant proportion of the global population. Abnormal host immune responses towards common antigens in the environment, coupled with genetic predisposition, form the matrix of atopic disease. Epidemiologic data arising from birth cohorts and cross-sectional studies around the world have significantly altered our understanding of the gene-environment interaction as it relates to allergic conditions and factors influencing the risk of developing disease. Our understanding of the biologic and immunologic mechanisms underpinning monogenic forms of the primary atopic disorders characterized by immune dysregulation has contributed towards targeted therapies. Ongoing research and applications of genomic testing to further characterize biological pathways may help pave the transition towards precision medicine for atopic disorders.

## References

[1] Perry R Braileanu G Palmer T Stevens P. The economic burden of pediatric asthma in the United States: literature review of current evidence. Pharmacoeconomics. (2019) 37(2):155–67. 10.1007/s40273-018-0726-2 30315512 PMC6386052

[2] Spergel JM. Epidemiology of atopic dermatitis and the atopic march in children. Immunol Allergy Clin North Am. (2010) 30(3):269–80. 10.1016/j.iac.2010.06.003 20670812

[3] Yang L Fu J Zhou Y. Research progress in atopic March. Front Immunol. (2020) 11:1907. 10.3389/fimmu.2020.01907 32973790 PMC7482645

[4] Lyons JJ Milner JD. Primary atopic disorders. J Exp Med. (2018) 215:1009–22. 10.1084/jem.20172306 29549114 PMC5881472

[5] Ogulur I Mitamura Y Yazici D Pat Y Ardicli S Li M Type 2 immunity in allergic diseases. Cell Mol Immunol. (2025) 22:211–42. 10.1038/s41423-025-01261-2 39962262 PMC11868591

[6] Strachan DP. Hay fever, hygiene, and household size. Br Med J. (1989) 299(6710):1259–60. 10.1136/bmj.299.6710.1259 2513902 PMC1838109

[7] Greenwood BM. Polyarthritis in Western Nigeria. I. Rheumatoid arthritis. Ann Rheum Dis. (1969) 28(5):488–96. 10.1136/ard.28.5.488 4186678 PMC1031236

[8] Stiemsma LT Reynolds LA Turvey SE Finlay BB. The hygiene hypothesis: current perspectives and future therapies. Immunotargets Ther. (2015) 4:143–57. 10.2147/ITT.S61528 27471720 PMC4918254

[9] Ege MJ Mayer M Normand AC Genuneit J Cookson WOCM Braun-Fahrlander C Exposure to environmental microorganisms and childhood asthma. N Engl J Med. (2011) 364:701–9. 10.1056/NEJMoa1007302 21345099

[10] Stein MM Hrusch CL Gozdz J Igartua C Pivniouk V Murray SE Innate immunity and asthma risk in Amish and Hutterite farm children. N Engl J Med. (2016) 375(5):411–21. 10.1056/NEJMoa1508749 27518660 PMC5137793

[11] Oluwole O Rennie DC Senthilselvan A Dyck R Afanasieva A Adamko DJ Asthma diagnosis among children along an urban-rural gradient. J Asthma. (2018) 55(11):1242–52. 10.1080/02770903.2017.1407335 29420108

[12] Von Mutius E. The “hygiene hypothesis” and the lessons learnt from farm studies. Front Immunol. (2021):12:635522. 10.3389/fimmu.2021.635522 33868259 PMC8044987

[13] McDade TW. Early environments and the ecology of inflammation. Proc Natl Acad Sci USA. (2012) 109(Suppl 2):17281–8. 10.1073/pnas.1202244109 23045646 PMC3477398

[14] Garn H Potaczek DP Pfefferle PI. The hygiene hypothesis and new perspectives—current challenges meeting an old postulate. Front Immunol. (2021) 12:637087. 10.3389/fimmu.2021.637087 33815389 PMC8012489

[15] Potaczek DP Alhamwe BA Miethe S Garn H. Epigenetic mechanisms in allergy development and prevention. Handb Exp Pharmacol. (2022) 268:331–57. 10.1007/164_2021_475 34223997

[16] McDade TW Georgiev AV Kuzawa CW. Trade-offs between acquired and innate immune defenses in humans. Evol Med Public Health. (2016) 2016(1):1–16. 10.1093/emph/eov033 26739325 PMC4703052

[17] Peroni DG Hufnagl K Comberiati P Roth-Walter F. Lack of iron, zinc and vitamins as a contributor to the etiology of atopic diseases. Front Nutr. (2023) 9:1032481. 10.3389/fnut.2022.1032481 36698466 PMC9869175

[18] Pfefferle PI Keber CU Cohen RM Garn H. The hygiene hypothesis—learning from but not living in the past. Front Immunol. (2021) 12:635935. 10.3389/fimmu.2021.635935 33796103 PMC8007786

[19] Frey SM Rowe RK Halterman JS. The prevalence of childhood asthma: interpreting falling rates in the context of shifting measurement and the COVID19 pandemic. Curr Opin Pulm Med. (2023) 29(3):197–201. 10.1097/MCP.0000000000000959 36917214 PMC10090305

[20] The International Study of Asthma and Allergies in Childhood (ISAAC) Steering Committee. Worldwide variations in the prevalence of asthma symptoms: the international study of asthma and allergies in childhood (ISAAC). Eur Resp J. (1998) 12(2):315–35. 10.1183/09031936.98.12020315 9727780

[21] Strachan D Sibbald B Weiland SK Ait-Khaled N Anabwani G Anderson HR Worldwide variations in prevalence of symptoms of allergic rhinoconjunctivitis in children: the International Study of Asthma and Allergies in Childhood (ISAAC). Pediatr Allergy Immunol. (1997) 8:161–76. 10.1111/j.1399-3038 9553981

[22] Beasley R, The International Study of Asthma and Allergies in Childhood (ISAAC) Steering Committee. Worldwide variation in the prevalence of symptoms of asthma, allergic rhinoconjunctivitis and atopic eczema: ISAAC. Lancet. (1998) 351:1225–32. 10.1016/S0140-6736(97)07302-9 9643741

[23] Mallol J Crane J von Mutius E Odhiambo J Keil U Stewart A The International Study of Asthma and Allergies in Childhood (ISAAC) phase three: a global synthesis. Allergol Immunopathol (Madr). 2013 41(2):73–85. 10.1016/j.aller.2012.03.001 22771150

[24] Pacheco-Gonzalez R Mallol J Solé D Brand PLP Perez-Fernandez V Sanchez-Solis M Factors associated with the time to the first wheezing episode in infants: a cross-sectional study from the International Study of Wheezing in Infants (EISL) 2016. NPJ Prim Care Resp Med. (2016) 26:15077. 10.1038/npjpcrm.2015.77 PMC4721498 26796896

[25] Asher MI Rutter CE Bissell K Chiang CY El Sony A Ellwood E Worldwide trends in the burden of asthma symptoms in school-age children: global asthma network phase I cross-sectional study. Lancet. (2021) 398:1569–80. 10.1183/13993003.02094-2020 34755626 PMC8573635

[26] Zanobetti A Ryan PH Coull B Brokamp C Datta S Blossom J Childhood asthma incidence, early and persistent wheeze and neighbourhood socioeconomic factors in the ECHO/CREW consortium. JAMA Pedatr. (2022) 176(8):759–67. 10.1001/jamapediatrics.2022.1446 PMC9127710 35604671

[27] Jartti T Gern JE. Role of viral infections in the development and exacerbation of asthma in children. J Allergy Clin Immunol. (2017) 140(4):895–906. 10.1016/j.jaci.2017.08.003 28987219 PMC7172811

[28] Bacharier L Cohen R Schweiger T Yin-Declue H Christie C Zheng J Determinants of asthma after severe respiratory syncytial virus bronchiolitis. J Allergy Clin Immunol. (2012) 130(1):91–100.e3. 10.1016/j.jaci.2012.02.010 22444510 PMC3612548

[29] Wu P Dupont WD Grifﬁn MR Carroll KN Mitchel AF Gebretsadik T Evidence of a causal role of winter virus infection during infancy in early childhood asthma. Am J Respir Crit Care Med. (2008) 178:1123–9. 10.1164/rccm.200804-579OC 18776151 PMC2588491

[30] Thomsen SF van der Sluis S Stensballe LG Posthuma D Skytthe A Kyvik KO Exploring the association between severe respiratory syncytial virus infection and asthma: a registry-based twin study. Am J Respir Crit Care Med. (2009) 179:1091–7. 10.1164/rccm.200809-1471OC 19286626

[31] Sigurs N Aljassim F Kjellman B Robinson PD Sigurbergsson F Bjarnason R Asthma and allergy patterns of 18 years after severe RSV bronchiolitis in the first year of life. Thorax. (2010) 65(12):1045–52. 10.1136/thx.2009.121582 20581410

[32] Wang G Han D Jiang Z Li M Yang S Liu L Association between early bronchiolitis and the development of childhood asthma: a meta-analysis. BMJ Open. (2021) 11:e043956. 10.1136/bmjopen-2020-043956 34049905 PMC8166632

[33] Munoz-Quiles C Lopez-Lacort M Diez-Domingo J Orrico-Sanchez A. Bronchiolitis regardless of its etiology and severity, is associated with increased risk of asthma: a population-based study. J Infect Dis. (2023) 228(7):840–50. 10.1093/infdis/jiad093 37015894 PMC10547461

[34] Navanandan N Jackson ND Hamington KL Everyman JL Pruesse E Secor EA Viral determinants of childhood asthma exacerbation severity and treatment response. J Allergy Clin Immunol Pract. (2025) 13(1):95–104. 10.1016/j.jaip.2024.09.020 39368548 PMC11717597

[35] Gern JE. How rhinovirus infections cause exacerbations of asthma. Clin Exp Allergy. (2015) 45(1):32–42. 10.1111/cea.12428 25270551

[36] Ilarraza R Wu Y Skappak CD Ajamian F Proud D Adamko DJ. Rhinovirus has the unique ability to directly activate human T cells *in vitro*. J Allergy Clin Immunol. (2013) 131(2):395–404. 10.1016/j.jaci.2012.11.041 23374267

[37] Skappak C Ilarraza R Wu YQ Drake MG Adamko DJ. Virus-induced asthma attack: the importance of allergic inflammation in response to viral antigen in an animal model of asthma. PLoS One. (2017) 12(7):e0181425. 10.1371/journal.pone.0181425 28742120 PMC5524340

[38] Ajamian F Ilarraza R Wu Y Morris K Odemuyiwa SO Moqbel R CCL5 persists in RSV stocks following sucrose-gradient purification. J Leukoc Biol. (2020) 108(1):169–76. 10.1002/JLB.4MA0320-621R 32450617

[39] Azad MB Konya T Maughan H Guttman DS Field CJ Chari RS Gut microbiota of healthy Canadian infants: profiles by mode of delivery and infant diet at 4 months. CMAJ. (2013) 185(5):385–94. 10.1503/cmaj.121189 23401405 PMC3602254

[40] Hoskinson C Medeleanu MV Reyna ME Dai DLY Chowdhury C Moraes TJ Antibiotics taken within the first year of life are linked to infant gut microbiome disruption and elevated atopic dermatitis risk. J Allergy Clin Immunol. (2024) 154(1):131–42. 10.1016/j.jaci.2024.03.025 38670232

[41] Donà D Barbieri E Daverio M Lundin R Giaquinto C Zaoutis T Implementation and impact of pediatric antimicrobial stewardship programs: a systematic scoping review. Antimicrob Resist Infect Control. (2020) 9(1):3. 10.1186/s13756-019-0659-3 31911831 PMC6942341

[42] Patrick DM Sbihi H Dai DLY Al Mamun A Rasali D Rose C Decreasing antibiotic use, the gut microbiota, and asthma incidence in children: evidence from population-based and prospective cohort studies. Lancet Respir Med. (2020) 8(11):1094–105. 10.1016/S2213-2600(20)30052-7 32220282

[43] Rider NL Truxton A Ohrt T Margolin-Katz I Horan M Shin H Validating inborn error of immunity prevalence and risk with nationally representative electronic health record data. J Allergy Clin Immunol. (2024) 153(6):1704–10. 10.1016/j.jaci.2024.01.011 38278184

[44] Poli MC Aksentijevich I Bousfiha A Cunningham-Rundles C Hambleton S Klein C Human inborn errors of immunity: 2024 Update on the classification from the International Union of Immunological Societies Expert Committee. Available online at: https://wp-iuis.s3.eu-west-1.amazonaws.com/app/uploads/2025/01/08170257/IEI-Final-Update-of-2024-Report-Jan-2025.pdf (accessed January 25, 2024). 10.70962/jhi.20250003 PMC12829761 41608114

[45] Vaseghi-Shanjani M Smith KL Sara RJ Modi BP Branch A Sharma M Inborn errors of immunity manifesting as atopic disorders. J Allergy Clin Immunol. (2021) 148:1130–9. 10.1016/j.jaci.2021.08.008 34428518

[46] Biggs CM Lu HY Turvey SE. Monogenic immune disorders and severe atopic disease. Nat Genet. (2017) 49(8):1162–3. 10.1038/ng.3925 28747751

